# Salvianolic Acid B Protects Against Fatty Acid-Induced Renal Tubular Injury via Inhibition of Endoplasmic Reticulum Stress

**DOI:** 10.3389/fphar.2020.574229

**Published:** 2020-12-15

**Authors:** Xiaoyi Mai, Xin Yin, Peipei Chen, Minzhou Zhang

**Affiliations:** ^1^ Department of Critical-care Medicine, Guangdong Provincial Hospital of Chinese Medicine, The 2nd Affiliated Hospital of Guangzhou University of Chinese Medicine, Guangzhou, China; ^2^ AMI Key Lab of Chinese Medicine in Guangzhou, Guangzhou, China

**Keywords:** salvianolic acid B, renal tubular injury, saturated fatty acid, apoptosis, ER stress

## Abstract

**Background/Aims:** Obesity-related kidney disease is associated with elevated levels of saturated free fatty acids (SFA). SFA lipotoxicity in tubular cells contributes to significant cellular apoptosis and injury. Salvianolic acid B (SalB) is the most abundant bioactive molecule from *Radix Salviae Miltiorrhizae.* In this study, we investigated the effect of SalB on SFA-induced renal tubular injury and endoplasmic reticulum (ER) stress, *in vivo* and *in vitro*.

**Methods:** C57BL/6 mice were assigned to five groups: a control group with normal diet (Nor), high-fat diet group (HFD), and HFD with three different SalB treatment doses, low (SalBL; 3 mg/kg), medium (SalBM; 6.25 mg/kg), and high (SalBH; 12.5 mg/kg) doses. SalB was intraperitoneally injected daily for 4 weeks after 8 weeks of HFD. After 12 weeks, mice were sacrificed and kidneys and sera were collected. Apoptosis and ER stress were induced in human proximal tubule epitelial (HK2) cells by palmitic acid (PA, 0.6 mM), tunicamycin (TM, 1 μg/ml), or thapsigargin (TG, 200 nM) *in vitro*.

**Results:** C57BL/6 mice fed a high-fat diet (HFD) for 12 weeks exhibited increased apoptosis (Bax and cleaved caspase-3) and ER stress (BIP, P-eIF2α, ATF4, CHOP, ATF6, IRE1α, and XBP1s) markers expression in the kidney, compared with control mice, which were remarkably suppressed by SalB treatment. *In vitro* studies showed that PA (0.6 mM) induced apoptosis and ER stress in cultured HK2 cells. SalB treatment attenuated all the adverse effects of PA. However, SalB failed to inhibit TM or TG-induced ER stress in HK2 cells.

**Conclusion:** The study indicated that SalB may play an important role in obesity-related kidney injury via mediating SFA-induced ER stress.

## Introduction

Obesity is one of the biggest health issues globally, in recent decades. It is considered a risk factor for developing chronic kidney disease (CKD) and kidney injury, which are characterized by structural remodeling of the kidney tissue, including glomerulomegaly, tubular apoptosis, and interstitial fibrosis (Wu and Kaufman, 2006; Alicic et al., 2013; Redon and Lurbe, 2015). Accumulation of saturated fatty acids (SFA) and high proteinuria levels have been implicated in obesity-related nephropathy pathogenesis (Praga and Morales, 2006; de Vries et al., 2014). Lipotoxicity mediated by increased intracellular SFA and their metabolites, accompanied with persistent proteinuria, can aggravate dysfunctional tubule response and epithelial apoptosis. This is followed by an inflammatory response and fibrosis, which contribute to nephropathy progression (Weinberg, 2006; Lindenmeyer et al., 2008).

The endoplasmic reticulum (ER) is the organelle responsible for protein, lipid, and steroid syntheses in cells (Bertolotti et al., 2000; Travers et al., 2000). Aggregation of aberrant proteins, including unfolded or misfolded proteins, results in ER stress. Increased intrarenal fatty acid levels can reportedly trigger oxidative and ER stress, which in turn activates the unfolded protein response (UPR) and cell apoptosis (Rutkowski and Kaufman, 2004; Xu et al., 2005; Zhang and Kaufman, 2006; Ron and Walter, 2007; Cao et al., 2012). The ER stress response is mediated by three sensors located at the ER membrane: protein kinase RNA (PKR)-like ER kinase (PERK), inositol-requiring protein-1α (IRE1α), and activating transcription factor 6 (ATF6) (Sano and Reed, 2013). The ER-resident chaperone, also known as binding immunoglobulin protein (BiP), assists in folding newly synthesized proteins, reduces misfolded protein accumulation, and promotes ER-associated protein degradation, which is induced by SFA (Ma and Hendershot, 2001; Xu et al., 2005; Li et al., 2016). Prolonged or severe ER stress activates PERK, which phosphorylates the α subunit of the eukaryotic translation initiation factor (eIF2α), resulting in the expression of activating transcription factor 4 (ATF4) and the transcription factor C/EBP homologous protein (CHOP) ultimately leading to cell apoptosis (Ma and Hendershot, 2001; Ron and Walter, 2007). IRE1α is another key molecule that regulates cell dysfunction. XBP1 is one of the RNAs targeted by the IRE1α RNase activity. Activated IRE1α cleaves the XBP1 mRNA to produce a transcriptionally active form (XBP1s), which contributes to restoring protein homeostasis and promotes cytoprotection (Han and Kaufman, 2016). Once released from BIP upon accumulation of misfolded proteins, ATF6 traffics to the Golgi complex to mediate the adaptive response to ER protein misfolding (Han and Kaufman, 2016).

*Radix Salviae Miltiorrhizae*, one of the most important traditional herbal medicines in China, has been widely used globally for various diseases, including cardiovascular and cerebrovascular diseases, Alzheimer’s disease, Parkinson’s disease, and renal diseases (Kang et al., 2004; Jiang R. W. et al., 2005; Zhou et al., 2005; Cheng, 2006; Lam et al., 2006; Wong et al., 2010; Zhang et al., 2010; Zhou et al., 2011). The principal bioactive components of *Radix Salviae Miltiorrhizae* all contribute to the therapeutic effects. Salvianolic acid B (SalB) is the major aqueous extract component of *Radix Salviae Miltiorrhizae* root, which exhibits multiple pharmacological activities such as antioxidative, cellular proliferation and differentiation, and anti-apoptotic effects (Soung et al., 2003; Shi et al., 2007; Tian et al., 2008; Wang et al., 2010; Zeng et al., 2010). Extensive pharmacological studies mainly showed that SalB has several beneficial effects on cardiovascular protection. The renoprotective activities of SalB, including anti-inflammatory, antifibrogenic, and antioxidative effects in renal cells (Li et al., 2017; Ma et al., 2017; He et al., 2020; Hu et al., 2020; Pang et al., 2020), were recently reported. Palmitic acid (PA), the most abundant SFA in blood, induces ER stress and is a proapoptotic factor in renal cells (Guo et al., 2007; Martinez et al., 2008; Wei et al., 2009; Sieber et al., 2010). This study aimed to investigate the effectiveness of SalB on renal tubular ER stress and apoptosis in high-fat diet-fed mice and PA-incubated HK2 cells.

## Materials and Methods

### Reagents and Antibodies

DMEM/F12 (catalog: 11320082) medium, trypsin (catalog: 15050057) and fetal bovine serum (FBS, catalog: 12484028) were obtained from Gibco (Carlsbad, CA, United States). Anti-VCAM-1 (catalog: ab134047) and anti-ICAM-1 (catalog: ab179707) antibodies were obtained from Abcam (Cambridge, United Kingdom). Anti-BCL-2 (catalog: 26593-1-AP), anti-BAX (catalog: 50599-2-lg), anti-caspase-3 (catalog: 66470-2-lg), anti-IRE1α (catalog: 27528-1-AP), anti-ATF6 (catalog: 24169-1-AP) and anti-XBP1s (catalog: 24868-1-AP) antibodies were obtained from Proteintech (Chicago, United States). Tunicamycin (catalog: 12819), anti-phospho-eIF2α (catalog: 3398), anti-eIF2α (catalog: 5324), anti-ATF4 (catalog: 11815), anti-BIP (catalog: 3177), and anti-CHOP (catalog: 2895) antibodies were obtained from Cell Signaling Technology (Boston, United States). Anti-β-actin (catalog: BM0627) was obtained from Boster Bio-engineering Limited Company (Wuhan, China). Hoechst 33258 (catalog: C1011) was obtained from Biyotime® Biotechnology (Shanghai, China). Salvianolic acid B (catalog: 115939-25-8 and MB6598) was purchased from CDMUST (Chengdu, China) and MEILUNBIO (Dalian, China). Palmitic acid (catalog: P0500), DMSO (catalog: D8418), Oil red O (catalog: O0625) and thapsigargin (catalog: T9033) were purchased from Sigma (Saint Louis, United States). *In Situ* Cell Death Detection Kit (catalog: 11684795910) was purchased from Roche (Basel, Switzerland). Cell Counting Kit-8 (CCK-8, catalog: CK04) was purchased from DOJINDO (Kyushu Island, Japan). Annexin V-PE reagent (catalog: KGA108-1) was purchased from KeyGEN BioTECH (Jiangsu, China).

### Animals and Experimental Methods

Eight-week-old male C57BL/6 mice (28 ± 3 g) were obtained from Guangdong Medical Laboratory Animal Center (Guangzhou, China) and housed at room temperature ranging 22–26 °C, with 55 ± 5% relative humidity, and a 12-h light/dark cycle for 1 week to adapt to their environment. Renal injury was induced by feeding the mice with a high-fat diet, containing 60% of total calories from fat for 12 weeks. The mice were assigned to five groups: a control group with normal diet (Nor), high-fat diet group (HFD), and HFD with three different SalB treatment doses, low (SalBL; 3 mg/kg), medium (SalBM; 6.25 mg/kg), and high (SalBH; 12.5 mg/kg) doses. SalB was intraperitoneally injected daily for 4 weeks after 8 weeks of HFD. The SalB treatment dosage in the mice was slightly adjusted basing on previous studies (Lou et al., 2017; Zhao et al., 2019). There were 10 mice in each group. Bodyweight and fasting blood glucose (BG) of the mice were measured at week 12, and the 24-h urine was collected in metabolic cages. After 12 weeks, all the mice were anesthetized with chloral hydrate, sera collected, and the kidneys were rapidly excised from mice for biochemical and histological examinations. All studies were conducted in accordance with the internationally accepted principles for laboratory animal use and care as found in the National Institutes of Health guidelines (NIH Publications No. 8023, revised 1978) and related ethical regulations of the Institutional Animal Care and Use Committee of Guangdong Province Hospital of Chinese Medicine.

### Biochemical Analysis

Cystatin C (Cys-C) and serum creatinine (S-Cr) of the mice were determined using Mouse Cystatin C ELISA Kit (catalog: CSB-E08386m, CUSABIO, Wuhan, China) and Creatinine Assay Kit (catalog: C011-2-1, Nanjing Jiancheng Bioengineering Institute, Nanjing, China), respectively. The fasting blood glucose was measured with a glucometer purchased from BAYER (Leverkusen, Germany), and the 24-h urine protein level was measured with BCA Protein Assay Kit (Thermo Fisher Scientific.

### H&E Staining, Triphosphate Nick-End Labeling Assay, and Immunohistochemistry

The kidney samples were fixed in 4% buffered paraformaldehyde, embedded in paraffin, then 5 μm sections were cut for hematoxylin and eosin (H&E) staining, terminal deoxynucleotidyl transferase-mediated deoxyuridine triphosphate nick-end labeling (TUNEL) assay and immunohistochemistry staining. The tissue sections were dewaxed and rehydrated, stained with hematoxylin and eosin, then observed under a light microscope (Nikon, Japan).

The TUNEL assay was performed as described previously (Liang et al., 2016). Briefly, sections were blocked with 5% bovine serum albumin (BSA, MPbio, California, United States) for 1 h at room temperature, then incubated with reagents from the TUNEL system kit, according to the manufacturer’s instructions. Green-labeled TUNEL-positive cells were observed under a fluorescence microscope at ×100 magnification.

Sections after dewaxed and rehydrated, were blocked by 5% BSA for 60 min. After blocking, the sections were incubated with primary antibodies against BIP at 4 °C overnight and then were incubated with anti-rabbit second antibodies (Boster, Wuhan, China) at room temperature for 1 h. The nuclei were stained with hematoxylin. The quantification of all raw images was performed using Image-Pro Plus 5.0 software.

### Analysis of Kidney Lipid Content

Frozen sections from the kidney tissues were infiltrated with 60% isopropanol for a few seconds, then stained with 0.3% oil red O for 5 min. The sections were rinsed with 60% isopropanol and washed thrice with PBS. The lipid content was analyzed under the Nikon microscope connected to a digital camera, with a macro conversion lens. The positively stained area was quantified using Image-Pro Plus 5.0 software.

### Cell Culture and Treatment

Immortalized human proximal tubular cell line (HK2) cells were obtained from ATCC (United States) and cultured as described previously (Mai et al., 2016). Briefly, the cells were cultured in DMEM/F12, containing 10% FBS, 1.20 g/L sodium bicarbonate, 100 U/ml penicillin, and 100 mg/ml streptomycin, at 37 °C in 5% CO_2_. PA used in this study was complexed with 0.3% BSA using ultrasound for 1 h before adding to cultured cells. HK2 cells were preincubated with SalB, 1 μM, 10 μM, or 100 μM for 1 h, followed by treatment with PA (0.6 mM), tunicamycin (1 μg/ml), or thapsigargin (200 nM) for another 24 h in fresh DMEM/F12, containing 0.3% BSA or not. The cells treated with vehicle (DMSO) and BSA were used as control. The SalB treatment dose in HK2 cells was slightly adjusted basing on previous studies (Wang et al., 2010; Pan et al., 2011).

### Cell Viability Assay

Cell viability was assessed using the Cell Counting Assay Kit-8 (CCK-8) assay. After SalB and PA treatments, HK2 cells were incubated with the CCK-8 reagent (10 µl) for 4 h and absorbance measured at 450 nm using an EPOH microplate reader (Bio Tek, Winooski, United States).

### Western Blotting

Western blotting was performed as described previously (Mai et al., 2016). Briefly, cultured HK2 cells and kidney cortex fragments were homogenized in RIPA (CST, United States) buffer, supplemented with protease inhibitor (Roche, Switzerland) and phosphatase inhibitor (Roche, Switzerland). The total protein content was measured using a BCA kit (Millipore, United States). Equal amounts of protein samples were separated on 8–12% SDS-PAGE, then transferred onto polyvinylidene fluoride (PVDF) membranes (Millipore, United States). Nonspecific binding was blocked with 5% (W/V) non-fat milk diluted with TBST (Tris–HCl 20 mM, NaCl 150 mM, 0.1% Tween 20, pH 7.5) at room temperature for 1 h. After washing with TBST, the blots were incubated with primary antibodies overnight at 4°C, then with the appropriate horseradish peroxidase-conjugated secondary antibodies for 1 h at room temperature. The blots were visualized with a ChemiDoc™ Touch Image System (Bio-rad, United States) and quantified using ImageJ or Image Lab software.

### Flow Cytometric Analysis

Apoptotic cells were determined by flow cytometry using an Annexin V-FITC/propidium iodide (PI) staining kit. Briefly, cells were trypsinized and resuspended at a density of 10^6^/ml. After washing twice with ice-cold phosphate buffer solution (PBS), the cells were resuspended in binding buffer and incubated with Annexin V-FITC/PI at room temperature in darkness for 15 min. Apoptotic cells were then evaluated on a NovoCyte instrument (ACEABIO, Belgium).

### RNA Isolation and Quantitative Real-time PCR

RNA isolation and quantitative real-time PCR were performed as described previously (Mai et al., 2016). Briefly, total RNA was extracted from mice kidney tissues using the TRIzol reagent (catalog: 15596018, Invitrogen, Carlsbad, United States). CDNA was synthesized using a PrimeScript RT reagent kit (catalog: RR047A, Takara Bio Inc., Tokyo, Japan). This was used for quantitative real-time PCR analysis using TB Green® Premix Ex Taq™ (catalog: RR420A, Takara Bio Inc., Tokyo, Japan), according to the manufacturers’ instructions. IL-1β, IL-6, and TNF-α mRNA were determined using the comparative cycle threshold method of relative quantitation, and GAPDH was used as an internal control. Specific primers were synthesized by Thermo Fisher Scientific (United States). Primer sequences for m-IL-1β: forward: 5′-TAC​CTA​TGT​CTT​GCC​CGT​GGA​G-3′; reverse: 5′-ATC​ATC​CCA​CGA​GTC​AC- AGAGG-3′; for m-IL-6: forward: 5′-CTG​CAA​GAG​ACT​TCC​ATC​CAG-3′; reverse: 5′-AGT​GGT​ATA​GAC​AGG​TCT​GTT​GG-3′; for m-TNF-α: forward: 5′-TTCCCAA- ATGGGCTCCCTCT-3′; reverse: 5′-GTG​GGC​TAC​GGG​CTT​GTC​AC-3′; for m-GAPDH: forward: 5′-TGA​CCT​CAA​CTA​CAT​GGT​CTA​CA-3′; reverse: 5′-CTT- CCC​ATT​CTC​GGC​CTT​G-3′.

### Statistical Analyses

All data were represented as mean ± SEM. Statistical analyses were performed by one-way ANOVA, followed by Bonferroni multiple comparison post hoc tests, with a 95% confidence interval. *p* values <0.05 were considered to be statistically significant.

## Results

### SalB Inhibits Tubular Injury in the Kidneys of High-Fat Diet Mice

Metabolic data from mice fed a Nor, HFD, and HFD with different SalB doses treatment (SalBL, SalBM, and SalBH) are shown in Table 1. The body weight, kidney weight, and serum glucose were significantly higher in HFD mice with or without SalB treatment when compared with Nor mice. Mice fed with high-fat diet showed reduced urine output and increased urinary protein compared to Nor group, and SalB treatment slightly improved urine output and protein excretion. The levels of two tubular injury markers, cystatin C and serum creatinine, were significantly increased in mice with high-fat diet, SalB treatment was associated with reduced levels of cystatin C and serum creatinine compared with HFD mice. These results indicated that SalB effectively improved renal function in HFD mice.

**TABLE 1:** Metabolic data of mice.

	Nor	HFD	SalBL	SalBM	SalBH
BW, g	30.27 ± 1.07	34.72 ± 0.94**	35.98 ± 1.51**	33.3 ± 0.86*	34.18 ± 1.12*
KW, g	0.40 ± 0.11	0.48 ± 0.20**	0.49 ± 0.17**	0.49 ± 0.12**	0.48 ± 0.14**
BG, mmol/L	5.59 ± 0.45	7.61 ± 0.35**	7.74 ± 0.23**	7.74 ± 0.41**	8.01 ± 0.36**
UO, ml/24 h	1.99 ± 0.21	1.32 ± 0.09**	1.27 ± 0.13**	1.48 ± 0.08*	1.83 ± 0.14^#^
URP, mg/24 h	7.39 ± 1.38	14.60 ± 2.72**	10.91 ± 1.35	12.98 ± 0.96	8.79 ± 1.03^#^
Cys-C, ng/ml	470.28 ± 25.71	536.15 ± 17.12*	506.40 ± 17.31	514.23 ± 29.34	466.09 ± 22.87^#^
S-Cr, μmol/L	11.52 ± 0.37	15.08 ± 0.55**	13.41 ± 0.63	13.02 ± 0.53^##^	12.06 ± 0.98^##^

Data are represented as means ± SEM (*n* = 6–10). Nor, mice fed with normal diet; HFD, mice fed with high-fat diet; SalBL, SalBM, or SalBH, mice fed with high-fat diet and treated with low dosage of SalB (3 mg/kg), medium dosage of SalB (6.25 mg/kg), or high dosage of SalB (12.5 mg/kg), respectively; BW, body weight; KW, kidney weight; BG, blood glucose; UO, urine output; URP, urinary protein; Cys-C, cystatin C; S-Cr, serum creatinine. *p < 0.05 and **p < 0.01, compared with Nor; ^#^ *p* < 0.05 and ^##^ *p* < 0.01, compared with HFD.

**FIGURE 1:**
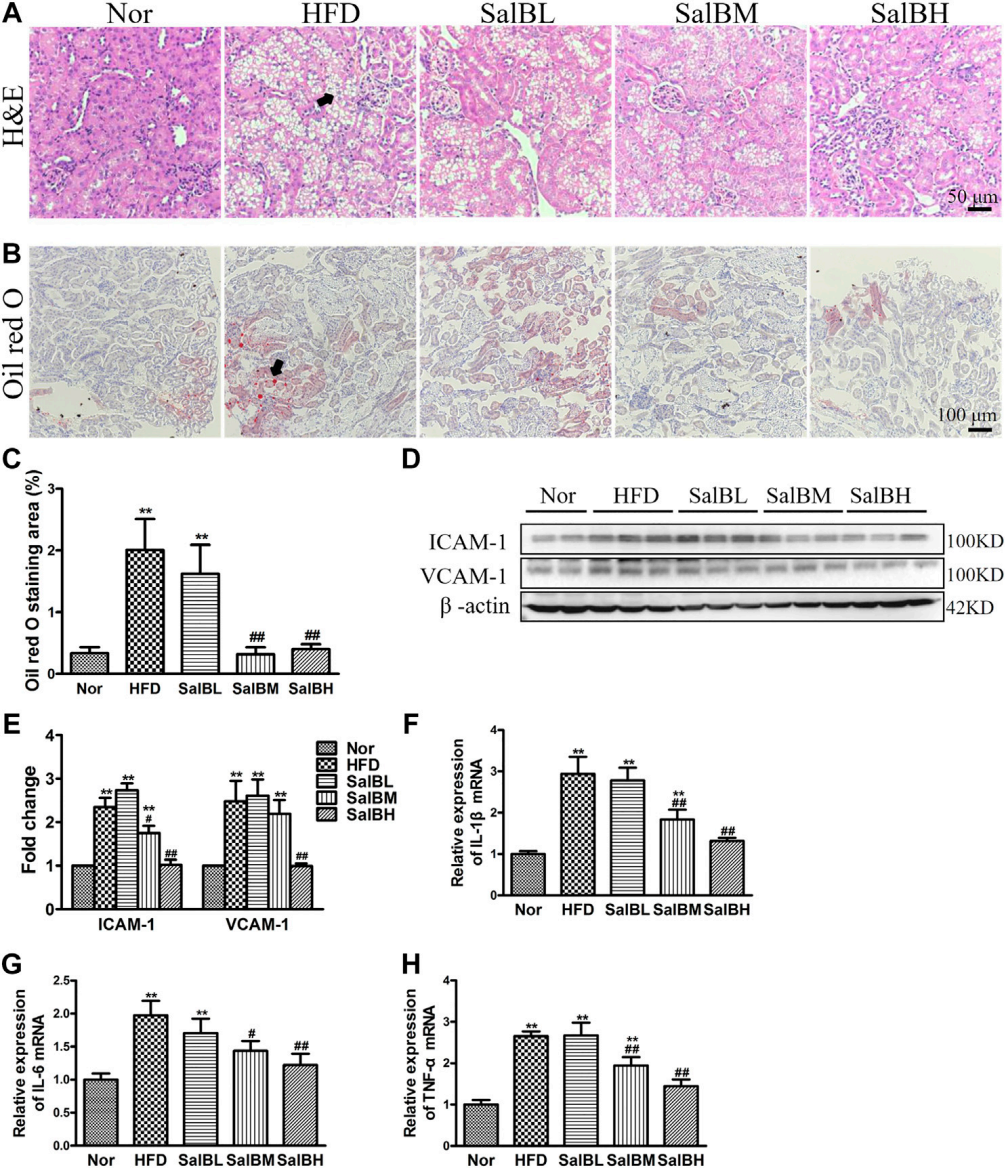
SalB inhibited tubular injury in the kidneys of HFD mice **(A)** H&E staining illustrated vacuolated proximal convoluted tubular cells (arrowheads) in HFD mice were attenuated by SalB treatment (magnification ×400) **(B)** The kidney lipid content in the mice was observed using oil red O staining (arrowheads) (magnification ×200). HFD was associated with increased lipid deposition in the kidney of the mice, which was ameliorated with SalB treatment **(C)** Quantitative analysis of Oil red O staining **(D,E)** Western blotting showed increased ICAM-1 and VCAM-1 expression in the kidney cortex of the HFD mice was ameliorated with SalB treatment. The corresponding quantifications were shown as well **(F,G,H)** Analysis of renal mRNA expression levels by quantitative real-time PCR for IL-1β, IL-6 and TNF-α in HFD-fed mice with or without SalB treatment. Nor, mice fed with normal diet; HFD, mice fed with high-fat diet; SalBL, SalBM, or SalBH, mice fed with high-fat diet and treated with low dosage of SalB (3 mg/kg), medium dosage of SalB (6.25 mg/kg), or high dosage of SalB (12.5 mg/kg), respectively. Data are represented as means ± SEM (*n* = 6). *p < 0.05 and **p < 0.01, compared with Nor; ^#^ *p* < 0.05 and ^##^ *p* < 0.01, compared with HFD.

We further observed the kidney morphology of HFD-induced obese mice. Hematoxylin and eosin (H&E) staining exhibited marked vacuoles in renal tubular cells in the cortex of HFD mice (Figure 1A). SalB treatment largely inhibited vacuolated tubular cell formation (Figure 1A). Likewise, Oil red O staining revealed that the HFD mice had increased kidney lipid accumulation in both tubules and glomeruli, which was significantly decreased by SalB treatment (Figures 1B,**C**). Intercellular cell adhesion molecule-1 (ICAM-1) and vascular adhesion molecule-1 (VCAM-1) are the major inflammatory adhesion molecules, which promote kidney injury development. Further western blotting experiments showed that ICAM-1 and VCAM-1 expressions were upregulated in the kidney cortex of the HFD mice, while suppressed by SalB treatment (Figures 1D,**E**). Moreover, renal mRNA expression levels of inflammatory markers, IL-1β, IL-6, and TNF-α were significantly increased in mice with high-fat diet but decreased after SalB treatment (Figures 1F–H). Taken together, these results indicated that SalB alleviated SFA-induced renal tubular injury.

### SalB Reduces Apoptosis in the Kidneys of High-Fat Diet-Fed Mice

**FIGURE 2:**
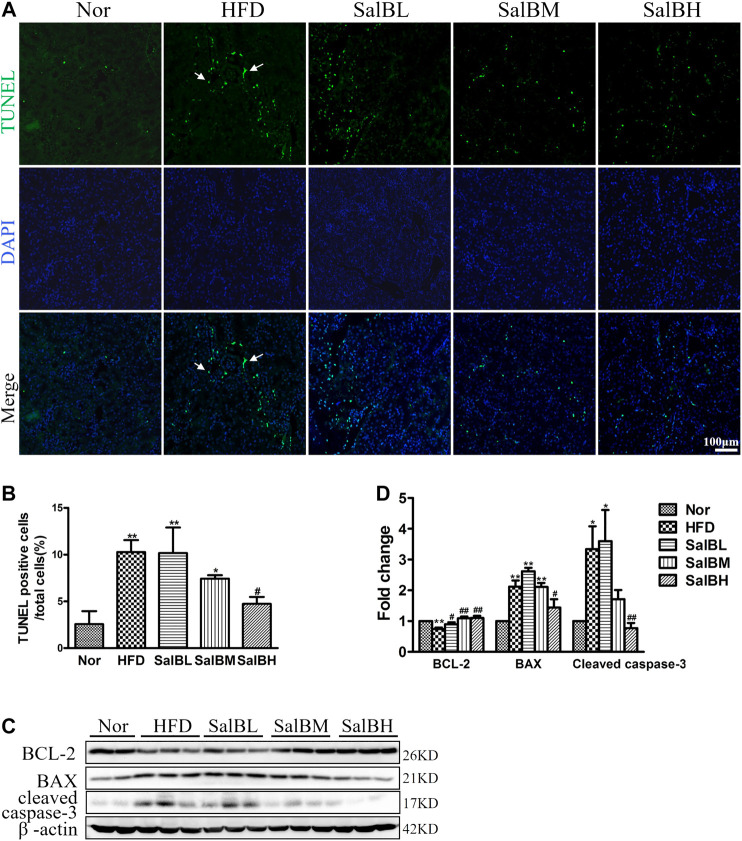
SalB suppressed HFD-induced apoptosis of renal tubular cells in mice **(A,B)** Apoptosis (arrowheads) induced by HFD in kidney cells was detected by TUNEL assays (green) (magnification ×200). The corresponding quantitative analysis of TUNEL assays was shown as well. Nuclei were counterstained with Hoechst 33258 (blue) **(C,D)** Western blotting and the corresponding quantitative analysis showed HFD-induced decrease in Bcl-2 and increase in BAX and cleaved caspase-3 protein levels in the kidney cortex, which were ameliorated with SalB treatment. Nor, mice fed with normal diet; HFD, mice fed with high-fat diet; SalBL, SalBM, or SalBH, mice fed with high-fat diet and treated with low dosage of SalB (3 mg/kg), medium dosage of SalB (6.25 mg/kg), or high dosage of SalB (12.5 mg/kg), respectively. Data are represented as means ± SEM (*n* = 6). *p < 0.05 and **p < 0.01, compared with Nor; ^#^ *p* < 0.05 and ^##^ *p* < 0.01, compared with HFD.

HFD was associated with a notably increased number of apoptotic cells in renal tubules, compared with the Nor mice (Figures 2A,B). The percentage of TUNEL-positive cells was significantly decreased by SalB treatment (Figures 2A,B). Immunoblotting was further performed to examine the expression of apoptosis-related genes BCL-2, BAX, and cleaved caspase-3. BCL-2, the anti-apoptosis protein, was attenuated in the HFD mice cortex, compared with the Nor mice. Nevertheless, SalB treatment restored the BCL-2 expression in the cortex of high-fat diet mice (Figures 2C,**D**). In contrast, the apoptosis-promoting genes BAX and cleaved caspase-3 were higher in the cortex of HFD mice than Nor mice. HFD-induced increase in BAX and cleaved caspase-3 protein expression in the kidney cortex of mice was suppressed after SalB treatment (Figures 2C,**D**).

### SalB Attenuates the Expression of Endoplasmic Reticulum Stress Markers in the Kidneys of High-Fat Diet-Fed Mice

**FIGURE 3:**
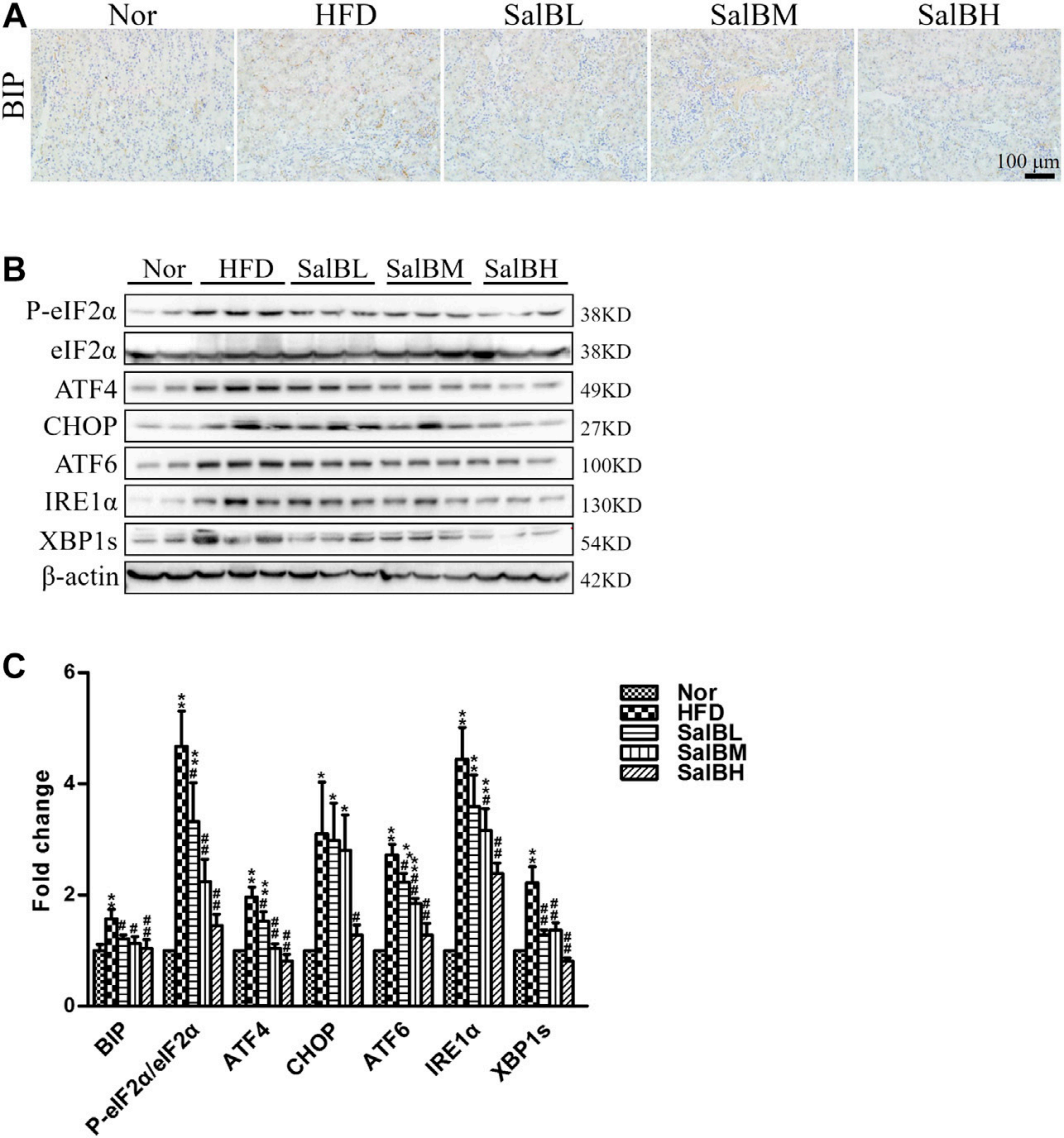
SalB attenuated HFD-induced ER stress in the kidney cortex of mice **(A,C)** Representative photomicrographs of BIP-stained kidney sections and the corresponding quantitative analysis **(B,C)** Protein levels of P-eIF2α, eIF2α, ATF4, CHOP, ATF6, IRE1α, and XBP1s in the kidney cortex were detected by western blotting. The corresponding quantitative analysis was shown as well. Nor, mice fed with normal diet; HFD, mice fed with high-fat diet; SalBL, SalBM, or SalBH, mice fed with high-fat diet and treated with low dosage of SalB (3 mg/kg), medium dosage of SalB (6.25 mg/kg), or high dosage of SalB (12.5 mg/kg), respectively. Data are represented as means ± SEM (*n* = 6–8). *p < 0.05 and **p < 0.01, compared with Nor; ^#^ *p* < 0.05 and ^##^ *p* < 0.01, compared with HFD.

To investigate whether ER stress is associated with HFD-induced apoptosis, we examined the activation of ER stress markers, including BIP, P-eIF2α, ATF4, CHOP, ATF6, IRE1α, and XBP1s, in the kidney cortex. Immunohistochemical analysis and western blotting demonstrated the expression of the above ER stress markers was significantly increased in the kidney cortex of HFD mice compared with Nor mice, while SalB treatment inhibited such an increase (Figures 3A–C). The findings above suggest that SalB performs a critical function in the ER stress-mediated apoptosis in renal tubular.

### SalB Inhibits Palmitic Acid-Induced Apoptosis in HK2 Cells

**FIGURE 4:**
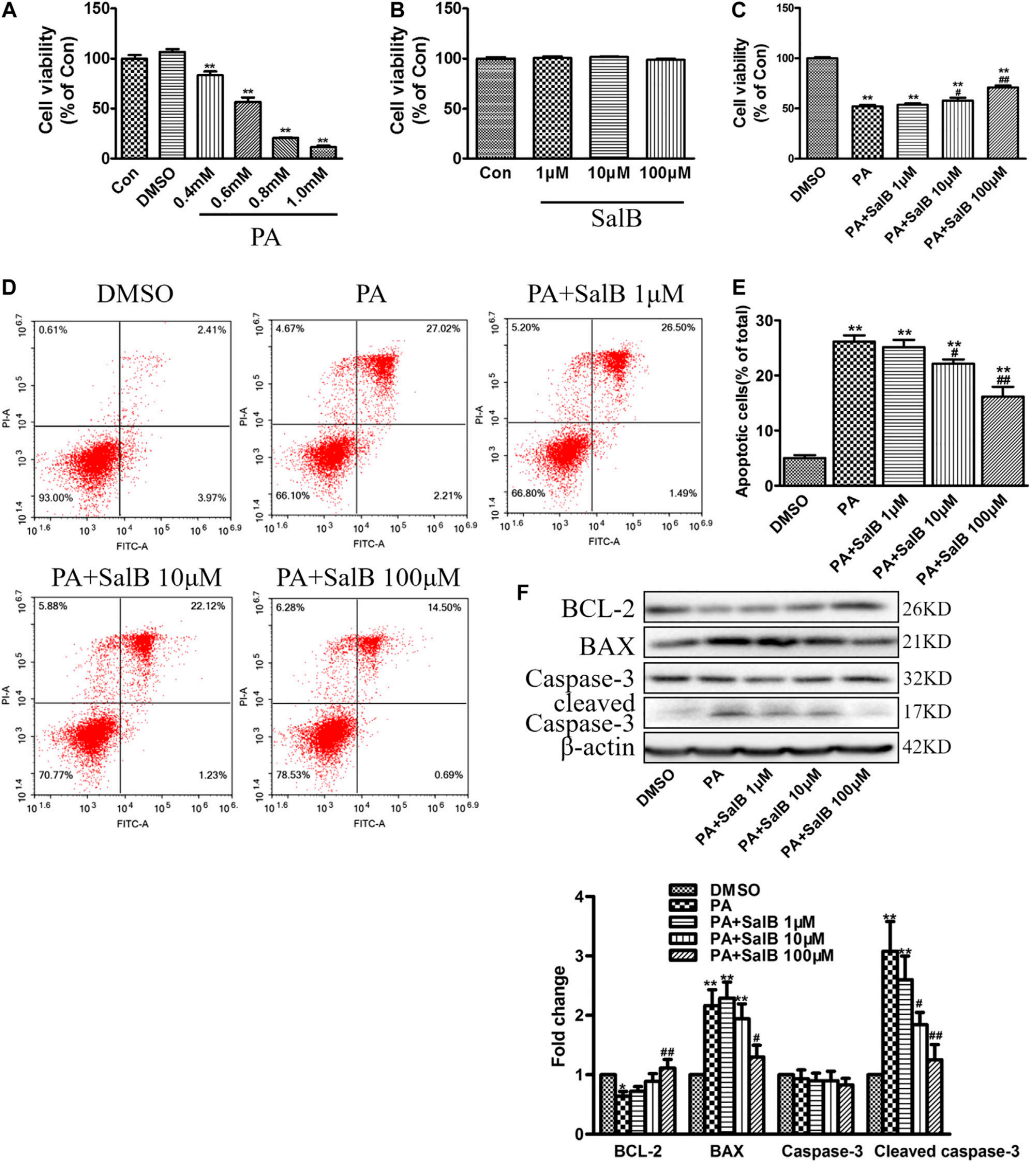
SalB inhibited PA-induced HK2 cells apoptosis **(A,B)** CCK-8 assay detection of HK2 cell viability after treatment with PA **(A)** or SalB **(B)** for 24 h **(C)** HK2 cells were preincubated with SalB, 1 μM, 10 μM, or 100 μM for 1 h, followed by treatment with 0.6 mM PA for another 24 h. CCK-8 assay showed SalB treatment in HK2 cells attenuated PA-induced cell death **(D,E)** Apoptosis ratios of HK2 cells were determined by flow cytometry, with Annexin-V/PI double staining **(F)** Western blotting and the corresponding quantitative analysis showed PA-induced decrease in BCL-2, and increase in BAX and cleaved caspase-3 protein levels in HK2 cells, which were ameliorated with SalB treatment. Data are represented as means ± SEM (*n* = 6). *p < 0.05 and **p < 0.01, compared with Con or DMSO; ^#^ *p <* 0.05 and ^##^ *P <* 0.01, compared with PA. Con, The control group; DMSO, DMSO treatment group; PA, palmitic acid treatment group; PA + SalB 1 μM, palmitic acid plus 1 μM SalB treatment; PA + SalB 10 μM, palmitic acid plus 10 μM SalB treatment; PA + SalB 100 μM, palmitic acid plus 100 μM SalB treatment.

We first examined the effect of PA on HK2 cells viability at various concentrations. CCK-8 assay showed a dose-dependent decrease in cell viability after PA stimulation for 24 h, compared with the controls (Figure 4A). The PA concentration 0.6 mM was employed for further experiments. Data from CCK-8 showed that SalB concentrations up to 100 mΜ are not cytotoxic (Figure 4B). However, SalB administration at 100 mΜ attenuated the PA-induced HK2 cytotoxicity (Figure 4C). The number of apoptotic cells after PA treatment was further evaluated using annexin V and PI double staining. Flow cytometry using Annexin V/PI showed that PA induced both HK2 cell apoptosis and necrosis (Figures 4D,**E**). Co-treatment with SalB inhibited apoptosis in PA-treated HK2 cells, dose-dependently (Figures 4D,**E**). Likewise, western blotting showed notably increased expression of BAX and cleaved caspase-3, but decreased BCL-2 expression in HK2 cells exposed to PA for 24 h (Figure 4F). SalB treatment decreased PA-induced BAX and cleaved caspase-3 levels, but restored BCL-2 expression (Figure 4F). Caspase-3 expression was unchanged in all treatments.

### SalB Suppresses Palmitic Acid-Induced Endoplasmic Reticulum Stress in HK2 Cells

**FIGURE 5:**
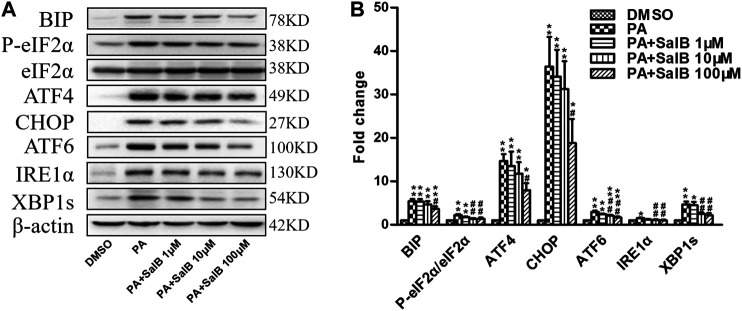
SalB attenuated PA-induced ER stress in HK2 cells **(A,B)** HK2 cells were preincubated with SalB, 1 μM, 10 μM, or 100 μM for 1 h, followed by treatment with 0.6 mM PA for another 24 h. Protein levels and the corresponding quantitative analysis of BIP, P-eIF2α, eIF2α, ATF4, CHOP, ATF6, IRE1α, and XBP1s in HK2 cells. Data are represented as means ± SEM (*n* = 4–9). *p < 0.05 and **p < 0.01, compared with DMSO; ^#^ *p <* 0.05 and ^##^ *p <* 0.01, compared with PA. DMSO, DMSO treatment group; PA, palmitic acid treatment group; PA + SalB 1 μM, palmitic acid plus 1 μM SalB treatment; PA + SalB 10 μM, palmitic acid plus 10 μM SalB treatment; PA + SalB 100 μM, palmitic acid plus 100 μM SalB treatment.

PA stimulates ER stress. Incubating with PA for 24 h increased the levels of ER stress markers in HK2 cells, including BIP, P-eIF2α, ATF4, CHOP, ATF6, IRE1α, and XBP1s (Figures 5A,B). The PA-induced upregulation of the above ER stress markers was attenuated by SalB treatment (Figures 5A,B). These results demonstrate that SalB protects HK2 cells from PA-mediated ER stress by inhibiting the PERK-eIF2α, as well as IRE1α/XBP1 and ATF6 pathways.

### SalB Does Not Inhibit Tunicamycin or Thapsigargin-Induced Endoplasmic Reticulum Stress in HK2 Cells

**FIGURE 6:**
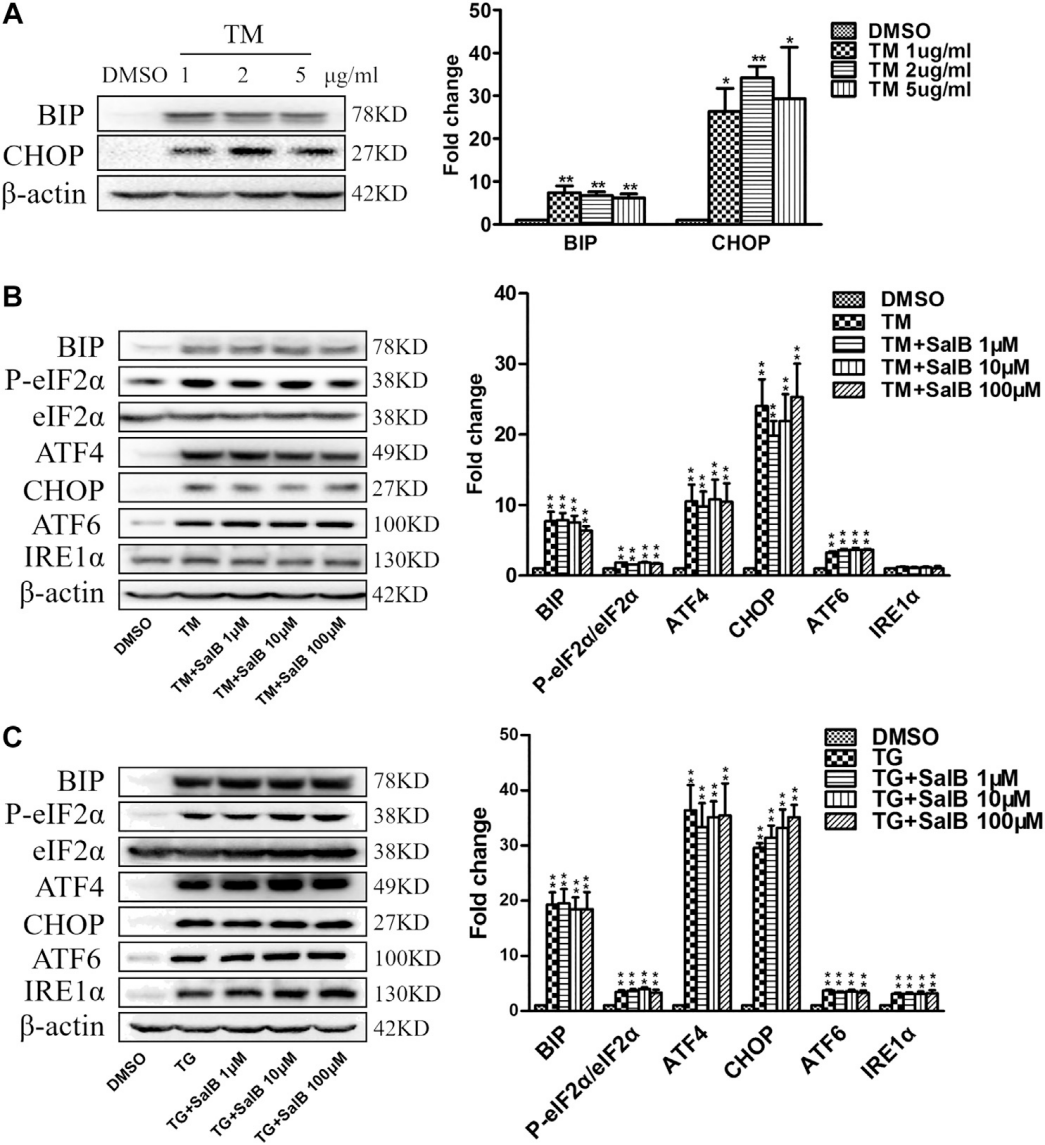
SalB did not inhibit tunicamycin or thapsigargin-induced ER stress in HK2 cells **(A)** Western blotting and the corresponding quantitative analysis showed that treatment with TM (1 μg/ml, 2 μg/ml, 5 μg/ml, respectively) for 24 h stimulated BIP and CHOP protein expression in HK2 cells **(B)** HK2 cells were preincubated with SalB, 1 μM, 10 μM, or 100 μM for 1 h, followed by treatment with 1 μg/ml TM for another 24 h. TM-induced upregulated expression of the ER stress markers BIP, P-eIF2α, eIF2α, ATF4, CHOP, and ATF6 in HK2 cells. Pretreatment with SalB did not attenuate the TM-induced ER stress. IRE1α expression was not increased after TM treatment in HK2 cells **(C)** HK2 cells were preincubated with SalB, 1 μM, 10 μM, or 100 μM for 1 h, followed by treatment with 200 nM TG for another 24 h. SalB did not decrease the expression of BIP, P-eIF2α/eIF2α, ATF4, CHOP, ATF6, and IRE1α induced by TG in HK2 cells. DMSO, DMSO treatment group; TM, tunicamycin treatment group; TG, thapsigargin treatment group; TM + SalB 1 μM, tunicamycin plus 1 μM SalB treatment; TM + SalB 10 μM, tunicamycin plus 10 μM SalB treatment; TM + SalB 100 μM, tunicamycin plus 100 μM SalB treatment; TG + SalB 1 μM, thapsigargin plus 1 μM SalB treatment; TG + SalB 10 μM, thapsigargin plus 10 μM SalB treatment; TG + SalB 100 μM,thapsigargin plus 100 μM SalB treatment. Data are represented as means ± SEM (*n* = 3–7). *p < 0.05 and **p < 0.01, compared with DMSO; ^#^ *p* < 0.05 and ^##^ *p* < 0.01, compared with TM or TG.

Next, we examined the effect of SalB inhibition on ER stress caused by other inducers, tunicamycin (TM) and thapsigargin (TG). Incubation of HK2 cells with TM (1, 2, and 5 μg/ml) for 24 h increased the ER stress markers BiP and CHOP (Figure 6A). There was no difference in ER stress stimulation among different TM concentrations. TM at 1 μg/ml was selected for the subsequent experiments. SalB was unable to reduce the expression of TM-induced ER stress markers, including BIP, P-eIF2α/eIF2α, ATF4, CHOP, and ATF6 (Figure 6B). The expression of IRE1α was unchanged among all treatments (Figure 6B). Consistent with previous studies, the expression of BIP, P-eIF2α/eIF2α, ATF4, CHOP, ATF6, and IRE1α was significantly increased in all TG-treated cells (Figure 6C). However, SalB treatment was unable to reduce these ER stress markers expression (Figure 6C). These results indicated that SalB is unlikely involved in TM or TG-induced ER stress in HK2 cells.

## Discussion

In this study, we found that SalB, a principal bioactive component of *S. miltiorrhiza*, is effective in protecting SFA-induced renal tubular injury and apoptosis, *in vivo* and *in vitro*. We further verified that SalB significantly attenuated SFA-associated ER stress in the kidney of HFD-fed mice or HK2 cells.

Accumulating evidence revealed that hyperlipidemia and hyperglycemia caused by obesity are associated with renal tubulointerstitial inflammation and injury (Park et al., 2007; Anders and Muruve, 2011; Li et al., 2016; Miyamoto et al., 2016; Yokoi and Yanagita, 2016). A previous study demonstrated that *S. Miltiorrhiza* extracts improved renal injury and regulated abnormal glycolipid metabolism in diabetic nephropathy (DN) rats (Xiang et al., 2019). The *S. miltiorrhiza* extracts were involved in some metabolic and the Wnt/catenin as well as TGF-β signaling pathways (Xiang et al., 2019). As the main active component of *S. miltiorrhiza*, SalB is considered to be effective on SFA-induced kidney injury.

Our results showed that the levels of serum cystatin C, serum creatinine, urine output, and urinary protein excretion, which are important indicators of renal injury, were significantly increased in mice with three months of HFD (Table 1). We also found tubular structure impairment and lipid accumulation in the proximal tubules in HFD-fed mice. SalB treatment showed effects in decreasing the levels of renal injury indicators and alleviating the tubular impairment. Renal tubular cells apoptosis is considered an important factor in renal disease development (Hauser and Oberbauer, 2002; Lau et al., 2012; Chen et al., 2018). The BCL-2 family, including antiapoptotic gene BCL-2, pro-apoptotic gene BAX, and caspase-3, are vital apoptosis-mediating proteases, which play important roles in apoptotic pathways. In this study, the renal injury in HFD-fed mice was associated with increased inflammation and apoptosis in the kidney cortex, which were inhibited by SalB treatment. SFA have been involved in the pathogenesis of obesity-induced tubulointerstitial injury (Yamahara et al., 2013). Of note, PA is the predominant circulating saturated fatty acid in obese animals (Shen et al., 2013). Our results confirmed that PA directly induced HK2 cell death and apoptosis, dose-dependently. SalB treatment in HK2 cells notably decreased PA-induced cell apoptosis. These data suggest SalB exerts its protective effect on renal injury by inhibiting renal tubular epithelial cells apoptosis.

ER stress is known to play a critical role in kidney disfunction (Kitamura, 2008; Taniguchi and Yoshida, 2015). Increased expression of ER stress markers in the kidney of renal ischemia/reperfusion (I/R) rats was observed (Abdallah et al., 2018). Additionally, fatty acid has been reported to induce ER stress in renal tubular epithelial cells (Lim et al., 2010). Prolonged or severe ER stress can induce tubular epithelial cells apoptosis. Several ER stress-associated pathways are involved in apoptosis induction. Three different signaling pathways mediated by the ER stress transducers PERK, IRE1α, or ATF6, have been identified. A previous study demonstrated that activation of the PERK-eIF2α-ATF4-CHOP pathway contributed to obesity-induced tubular epithelial cell apoptosis (Gun et al., 2017). EIF2α phosphorylation induced by PERK activation upregulates ATF4, then activates the amino acid response element and induces CHOP expression (Ma et al., 2002). This process ultimately upregulates the ratio of BAX/BCL-2 and initiates cellular apoptosis. The IRE1α/XBP1 pathway also plays an important role in regulating cell dysfunction (Sano and Reed, 2013). IRE1α mediates XBP1 mRNA expression to produce the potent transcriptional activator XBP1, which activates genes encoding ER chaperones and decreases ER stress (Zhuang and Forbes, 2014). Activated ATF6 is transferred to the Golgi where its cytosolic domain is cleaved to form an active transcriptional factor that mediates expression of several components adapting response to ER protein misfolding (Sano and Reed, 2013). SalB has been found to improve hepatic ER stress in ob/ob mice via inhibiting the PERK and IRE1α pathways (Shi et al., 2020). Additionally, doxorubicin-induced ER stress led to apoptotic damage in the heart tissues of the mice, which was attenuated by SalB treatment (Chen et al., 2016; Chen et al., 2017). These previous studies reveal an important role of SalB in ER stress. Thus, we hypothesized that SalB alleviated proximal tubular cell apoptosis and ameliorated functional abnormalities via abrogating SFA-mediated ER stress. The present study demonstrated that expression levels of BIP, P-eIF2α, ATF4, CHOP, ATF6, IRE1α, and XBP1s were increased in the kidney of the HFD-fed mice, which were alleviated by SalB treatment. Additionally, our data indicated that PERK, as well as IRE1α and ATF6pathway-induced ER stress were activated by PA in the proximal tubule cells, which was significantly inhibited by SalB treatment. However, the findings of the current study showed that SalB lost its effect on ER stress induced by tunicamycin or thapsigargin, two typical chemical inducers, indicating that SalB is especially associated with PA-induced ER stress in proximal tubule cells.

Recent studies demonstrated that lipid accumulation occurs in the kidney after a high-fat caloric exposure (Jiang T. et al., 2005; Decleves et al., 2014). SalB has been verified to affect the lipid metabolism both *in vivo* and *in vitro*. For instance, lipid uptake and lipid accumulation in macrophages were significantly reduced after SalB treatment (Bao et al., 2012). Another study reported that SalB dramatically improved lipid metabolites in diabetic rats (Huang et al., 2015). Our findings revealed that SalB treatment attenuated lipid accumulation in kidneys of the HFD-fed mice, suggesting the involvement of SalB in maintaining the homeostasis of kidney cells during increased SFA overload. The above results indicate that the potential mechanism by which SalB inhibits SFA-induced ER stress is related to its regulation of lipid metabolism in proximal tubule cells. However, this study did not clearly define the detailed mechanisms of the effects of SalB on SFA-mediated ER stress. Further investigations should be performed to thoroughly elucidate the beneficial effects of SalB on obesity-induced kidney damage.

Increased SFA levels contribute to lipotoxicity in renal tubular and glomerular cells, which leads to obesity-related glomerulopathy and diabetic nephropathy. It is crucial to explore new therapies for patients with obesity-related kidney damage. Clinical research has revealed the therapeutic effects of *S. miltiorrhiza* in DN patients (Nie and Li, 2018). As shown in the study, *S. miltiorrhiza* injection, with telmisartan, has beneficial synergistic effects for DN patients via reversing the increase in fibronectin and collagen IV, which attenuates the hyperglycemic state and ultrastructure changes of the glomerular basement membrane (Nie and Li, 2018). This current research mainly focused on the ameliorative effect of SalB on renal tubular function. In this study, we demonstrated for the first time that SalB inhibits SFA-induced proximal tubular cell apoptosis by suppressing ER stress. The protective effects of SalB on ER stress were involved in lipid metabolism regulation in the proximal tubular cells. These findings provide promising therapeutic targets for obesity-induced kidney injury. Nevertheless, more clinical studies are needed to validate them.

## Conclusion

In summary, this study unmasks the critical role of SalB in obesity-associated renal disease development. SalB reduced SFA-induced apoptosis and ER stress of renal tubular cells; thus, suppressing kidney injury progression.

## Data Availability Statement

The raw data supporting the conclusions of this article will be made available by the authors, without undue reservation.

## Ethics Statement

The animal study was reviewed and approved by Institutional Animal Care and Use Committee of Guangdong Province Hospital of Chinese Medicine.

## Author Contributions

XM and MZ designed the research and edited the manuscript; XM and YX performed experiments; PC carried out the data analysis.

## Funding

This work was supported by the National Natural Science Foundation of China (81703593).

## Conflict of Interest

The authors declare that the research was conducted in the absence of any commercial or financial relationships that could be construed as a potential conflict of interest.
